# High-Level Executive Functions: A Possible Role of Sex and Weight Condition in Planning and Decision-Making Performances

**DOI:** 10.3390/brainsci12020149

**Published:** 2022-01-24

**Authors:** Francesca Favieri, Giuseppe Forte, Mariella Pazzaglia, Eunice Y. Chen, Maria Casagrande

**Affiliations:** 1 Dipartimento di Psicologia, Università di Rome “Sapienza”, Via dei Marsi 78, 00185 Rome, Italy; g.forte@uniroma1.it (G.F.); mariella.pazzaglia@uniroma1.it (M.P.); 2 Body and Action Lab, IRCCS Fondazione Santa Lucia, Via Ardeatina 306, 00179 Rome, Italy; 3 Department of Psychology, Temple University, Weiss Hall 1701 N 13th St, Philadelphia, PA 19122, USA; Eunice.chen@temple.edu; 4 Dipartimento di Psicologia Dinamica, Clinica e Salute, Università di Rome “Sapienza”, Via Degli Apuli 1, 00185 Rome, Italy; maria.casagrande@uniroma1.it

**Keywords:** decision making, planning, executive functions, weight condition, gender differences

## Abstract

Evidence indicates an association between executive functioning and increased weight, with different patterns ascribed to individual differences (sex, age, lifestyles). This study reports on the relationship between high-level executive functions and body weight. Sixty-five young adults participated in the study: 29 participants (14 males, 15 females) in the normal weight range; 36 participants (18 males, 18 females) in the overweight range. The Iowa Gambling Task (IGT) and Tower of London Task were administered to assess decision making and planning. Planning did not differ in individuals in the normal-weight and overweight groups, and no difference emerged between females and males. However, normal and overweight males and females had different patterns in decision making. On the long-term consequences index of the IGT, females reported lower scores than males. Males in the overweight range had a lower long-term consequences index on the IGT than normal-weight males, while this pattern did not emerge in females. These findings suggest that decision-making responses may differ in the overweight relative to healthy weight condition, with a different expression in males and females. This pattern should be considered in weight loss prevention strategies, possibly adopting different approaches in males and females.

## 1. Introduction

Excessive weight is a risk factor for many chronic conditions (e.g., hypertension [1], diabetes [2], cardiovascular disorders [3]) and is related to psychological disorders (including anxiety and depression [4,5]), cognitive dysfunctions [6], and a general impairment of well-being and quality of life [7]. Over the last few decades, the prevalence of overweight conditions and obesity has substantially increased worldwide [8,9]. Maladaptive eating behavior is one of the main causes of body weight increase, and it appears to be influenced by many psychological (e.g., mood, impulsivity [10]; emotion regulation [11]; attentional bias [12]) and environmental (e.g., food availability, social pressure [13]) factors. Moreover, evidence has highlighted an association between impairments in executive functions and weight increase across the life span (for a review: [6,14]), especially in the individuals affected by obesity (i.e., body mass index (BMI) above 30 kg/m^2^). Cross-sectional studies have shown that poorer performance in executive functioning tasks is more likely to be associated with obesity than normal-weight status [6,8]. Longitudinal studies have observed an association between cognitive impairment and weight gain and between poorer performance on executive tasks and weight loss failure [15,16,17]. Most studies on the executive problems associated with obesity have focused on less complex executive functions such as working memory, inhibition, and set-shifting [6]. However, some studies have investigated the relationship between obesity and more complex executive functions, such as decision making and planning, showing impairment in these functions in association with obesity (BMI > 30 e.g., [15,18,19]). Moreover, there are also conflicting findings in the literature examining the relationship between executive functioning and overweight conditions (i.e., BMI between 25 and 30 kg/m^2^). Studies are poor and report discrepancies, although investigations on this topic could provide insight into the genesis of the association between these variables. For this reason, studying the association between overweight status and high-level executive functions could be relevant. Another important aspect of the relationship between executive functioning and weight is the relationship between executive functioning and biological sex. Previous studies have shown how females and males show different eating behavior patterns [20]; furthermore, the prevalence and incidence of overweight and obesity differ between sexes [21]. Females, compared to males, show a higher sensitivity to environmental food cues, which may account for the higher prevalence of obesity in the female population [21]. Moreover, some authors have highlighted a certain functional difference in executive functioning when gender differences are considered, especially in higher-level executive functions (e.g., decision making), which are more influenced by secondary factors such as metabolic, hormonal, and autonomic factors [22].

A recent meta-analysis by Rotdge and colleagues [8] analyzed the association between obesity and decision making as assessed by the Iowa Gambling Task (IGT; [23]), a gold standard task for assessing decision-making abilities under ambiguous conditions where individuals lack complete knowledge of the different options available. This meta-analysis reported an association between obesity and decision making under uncertain conditions [8]. Furthermore, the meta-analysis showed that poorer decision making was associated with the failure of a weight-loss program. Poorer performance on decision-making tasks in individuals with obesity [24,25,26] is associated with difficulty in making adaptive decisions in daily life that is related to the overeating that leads to weight gain. However, the few studies evaluating the association between IGT performance and overweight (BMI between 25 and 30 kg/m^2^) did not confirm this finding in obesity [15,27], indicating a possibly different pattern in less severe conditions of body weight. Moreover, the studies did not consider possible sex differences.

This study aims to provide new evidence about the relationship between executive functions and body weight by analyzing more complex and less investigated (i.e., decision making and planning) executive functions in overweight conditions, differentiating female and male executive patterns. Specifically, the present study investigated the association between body weight and decision making and planning in a sample of healthy individuals included in the normal weight to overweight continuum, without eating disorders, medical or psychopathological conditions, and severe obesity (BMI > 35 kg/m^2^). Given previous findings in adults with obesity [24,27,28], poorer performance in decision-making tasks under risk (IGT; [29]) is expected for overweight subjects compared to those with normal weight. According to the hypothesis of a general executive impairment related to overweight status [6], we expected lower planning functioning in the overweight subjects of our study [30]. Moreover, considering the possible sex differences [20,31], we expected different patterns in decision making and planning between females and males in the normal weight and overweight ranges.

## 2. Materials and Methods

### 2.1. Participants

Six-five participants (32 males and 33 females; mean age: 24 years SD = 3) voluntarily took part in the study. Specifically, 29 participants (14 males; 15 females) reported a BMI under the threshold of overweight (25 kg/m^2^) and were classified as normal weight; 36 participants reported a BMI beyond the threshold of overweight (18 males; 18 females). Table 1 details the characteristics of the sample, and Table 2, the group scores on the executive functioning tasks.

The study included the participants if they did not present an eating disorder diagnosis, food allergies, severe obesity, chronic medical diseases, or any psychological conditions (e.g., anxiety, depression).

### 2.2. Outcomes

#### 2.2.1. Demographic and Clinical Information

A semi-structured interview was adopted to collect the main demographic information of each participant (gender, age, years of education) and medical and clinical history.

#### 2.2.2. Executive Functions

*Decision Making*

Decision making was assessed using a computerized version of the Iowa Gambling Task (IGT; [29]), completely superimposable on the original version [32].

Apparatus: the task was administered via E-Prime 2.1 software (Psychology Software Tools Inc., Pittsburgh, PA, USA) on a personal computer equipped with a 15-inch monitor. The responses were enabled by four keys of the computer keyboard.

Stimuli: Four decks of cards (“A”, “B”, “C”, and “D”) with a red cover in the back and a Joker in the front constituted the stimuli on a green background [29].

Procedure: each card in the decks was associated with a win or a loss. The decks differed in the frequency and number of wins and losses. Decks A and B were considered disadvantageous, with large short-term wins ($100) but long-term losses. Deck A was associated with more frequent loss but less plentiful than deck B. Overall, decks A and B led to a loss of $250 for every 10 cards drawn. Decks C and D were more advantageous, although characterized by a small short-term payout ($50 each). The two decks differed in the frequency and magnitude of the loss. Deck C had more frequent but lower losses than deck D. Every 10 cards drawn on these decks resulted in a win of $500 with a loss of $250. The amount of money won (written in green) and lost (written in red) was shown for each trial, and the total budget was indicated during the overall task duration. Each participant started with a $2000 credit and was informed that some decks were more advantageous than others.

The participant had to press one of the four keys in the keyboard, corresponding to the deck they intended to choose. The test ended automatically after the hundredth selection (100 trials). The locations of the losses in this experiment were adopted by Bechara et al. [29]. The learning of long-term consequences (LTC) and the bias of infrequent loss (IFL) indices were calculated. The LTC was calculated by subtracting the number of disadvantageous choices from the number of advantageous choices ((C + D) − (A + B)). The IFL was calculated by subtracting the frequent-loss deck choices from the infrequent-loss deck choices ((A + C) − (B + D)). Higher scores in both indices indicated a better decision-making function.

**Figure 1:**
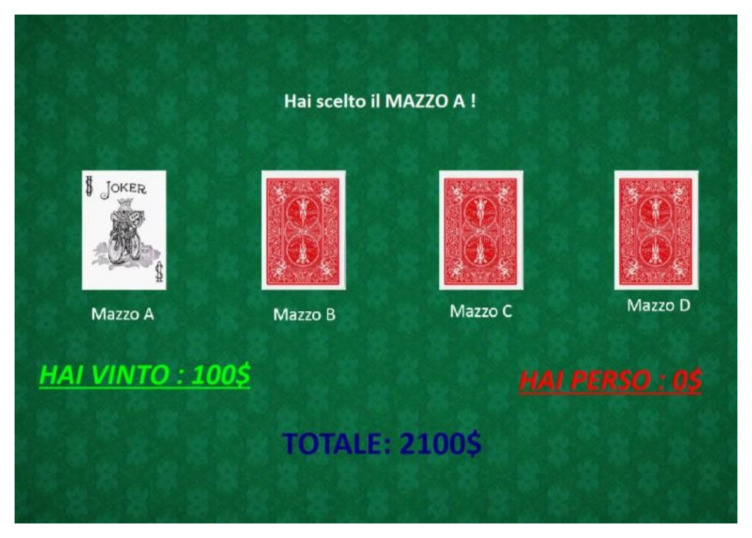
Example of the IGT procedure.

An example of the IGT procedure is shown in Figure 1.

*Planning*

Planning abilities were assessed by a computerized version of the Tower of London task [33,34].

Apparatus: the task was administered via Pebl 2.1 software [33] computer software retrieved from http://pebls.sf.net; GNU General Public License, accessed on 6 January 2021.) on a personal computer equipped with a 15-inch monitor. Participants responded using a computer mouse.

Stimuli: on the top of the screen, three colored discs (blue, green, red) were located on a structure with three vertical sticks in a predefined order. The same frame was presented at the bottom of the screen but with movable discs.

**Figure 2:**
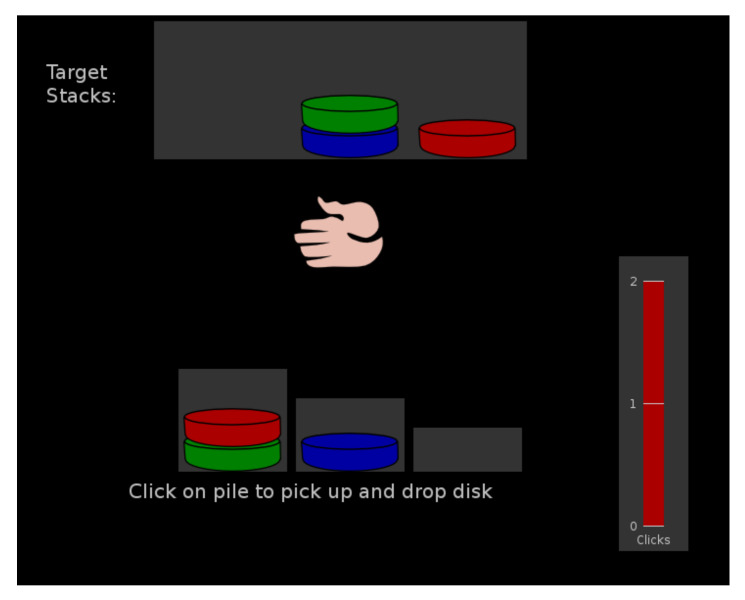
Example of the TOL procedure.

Procedure: the participant must order the discs one by one to recreate the configuration shown at the top of the screen, employing a maximum of 12 trials. The whole sequence must be carried out mentally before being performed. For each trial, only a predetermined number of movements can be made, and the number of available movements was shown on a vertical bar at the side of the screen. A total score was calculated by the Pebl program, considering the number of trials correctly completed in the minimum possible moves. A lower total score indicated lower planning performances. An example of the TOL procedure is shown in Figure 2.

### 2.3. Apparatus

A digital balance was used to assess the weight of each participant (kg), and a wall-mounted anthropometer was adopted to measure the height (m). BMI was calculated by dividing weight by height (in meters squared). The WHO criteria were adopted to classify BMI (WHO, 2020). Waist and hip circumferences were measured by a tape measure. The waist-to-height ratio (W/Hr; [34]) and body adiposity index (BAI = ((hip circumference)/((height)1.5) – 18)); [35]) were calculated as alternative indices of body weight. A digital sphygmomanometer was used to measure the participants’ systolic and diastolic blood pressure, considered as confounding variables in the analysis.

### 2.4. General Procedure

Written informed consent was administered to each participant before the evaluation. The research was conducted according to the Helsinki Declaration, and it was approved by the Local Ethics Committee (Department of Dynamic and Clinical Psychology and Health Studies—“Sapienza” the University of Rome; cod. 0000450-15 April 2019). Each participant was tested in a silent, dimly illuminated room with a comfortable temperature. Before the experimental session, where the IGT and TOL were randomly administered, the aims of the study were explained to the participant, and the semi-structured interview was administered.

### 2.5. Data Analysis

The descriptive analyses were calculated, considering the sex (males and females) and the weight condition (normal weight, overweight). Univariate analyses of variance (ANOVAs) were carried out to control participants’ differences in age, years of education, and physiological measures (see Table 1).

Mixed ANOVAs were carried out to assess the differences between the groups, considering sex and body weight condition, in the LTC and IFL indices of the five blocks of the IGT and the mean score. To assess the planning performances in the groups, an ANOVA on the total score of the TOL was carried out.

## 3. Results

**Figure 3:**
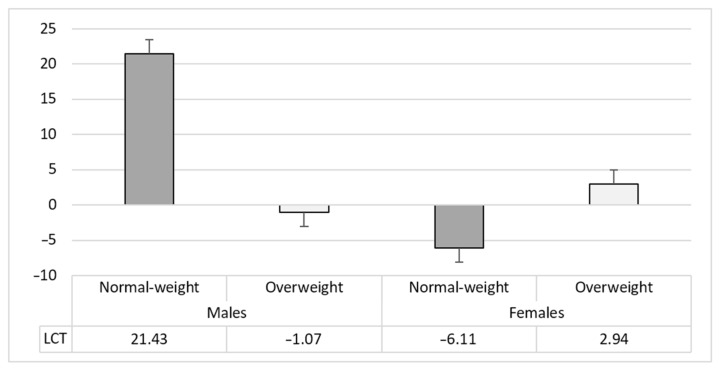
Mean and Std.Error of the LCT index (Sex × Weight Condition interaction).

Considering the LTC index of the IGT, the ANOVA showed a significant effect of Sex (F_1,61_ = 4.99; *p* = 0.03; ƞ^2^_p_ = 0.07), with females reporting lower LTC scores than males. The significant Sex × Weight Condition (F_1,61_ = 8.97; *p* = 0.004; ƞ^2^_p_ = 0.13) interaction highlighted that males with overweight showed lower LTC scores than normal-weight males (mean difference = −22.50; *t* = −2.89; *p* = 0.03). Moreover, normal-weight females reported lower LTC than normal-weight males (mean difference = −27.54; *t* = 3.69; *p* = 0.003). No other differences emerged (*p* > 0.08) (see Figure 3).

Considering the IFL index of the IGT no main effects of Sex (F_1,61_ < 1.00; *p* = 0.99) and Weight condition (F_1,61_ < 1; *p* = 0.94) were present, nor was the Sex x Weight Condition interaction significant (F_1,61_ < 1; *p* = 0.74).

The ANOVA on the Global score of the TOL did not show significant differences between groups for the main effects of Sex (F_1,61_ < 1; *p* = 0.67), the Weight condition (F_1,61_ = 2.10; *p* = 0.15), or the Sex x Weight condition (F_1,61_ < 1; *p* = 0.94) (see Table 2).

## 4. Discussion

Previous studies have confirmed an association between executive functions and both maladaptive eating behavior [36] and excessive body weight [6,37]. However, a large portion of these studies focused on basic executive functions (i.e., inhibition, working memory, shifting), while the association between more complex executive functions (i.e., problem solving, decision making, planning) and weight status has been poorly analyzed. Moreover, the research on this topic has focused on obesity and not on the earliest stages of weight gain, i.e., individuals in the overweight range. This study is one of the first to analyze the relationship between weight status in healthy individuals of normal weight to overweight ranges and higher executive functioning, focusing on planning and decision making. The present study assumed that planning and decision making, which involve different cognitive mechanisms and neural substrates aimed at controlling goal-directed behaviors, could affect (or be affected by) weight gain. We also assessed the role of self-reported biological sex. Biological sex differences in reward-based decision making have been demonstrated [31,38], suggesting that females tend to focus on short-term reward outcomes, whereas males focus on long-term decision outcomes. Differences in dopaminergic and serotoninergic activity may influence the different risk-taking decision-making performances between males and females [39]. Sex has also been reported to have played a role in studies on planning, with better performance in males than females in tasks involving planning abilities [40]. Moreover, different patterns in eating behavior were reported by females and males, influencing the differences in maladaptive eating behaviors that allow people to overeat. The interrelation between overweight status and sex on tasks assessing high-level executive functions could explain the risk of overweight conditions.

According to Damasio’s somatic marker hypothesis [41], some authors [8] have hypothesized a possible association between decision-making differences and eating behaviors associated with obesity. Decision making overlaps with some aspects of reward sensitivity [42], and it is characterized by the tendency to assign values and probabilities to behavioral patterns aimed at a specific outcome (e.g., select the more convenient option among several ones). Some specific endogenous (psychological characteristics, hormonal balance) and exogenous factors (social influences, relationships, environmental stimuli), which influence reward sensitivity to food stimuli, could generate overeating behavior [43]. Hypersensitivity to immediate reward and the failure to generate appropriate responses to visceral signals (e.g., gut activity) [44] or the presence of impulse-control problems [6,8] may account for individual differences in reward sensitivity leading to differences in decision making.

Although studies on obesity demonstrate an association between severe body adiposity and impairment in decision making (for a review, see [6,8]), studies that have analyzed the relationship between decision making and less severe overweight status in healthy populations have not confirmed this association [15,27]. This suggests that obesity models in which overeating is theorized to be associated with poorer decision making [45] may be true for overweight individuals.

Generally, the results of our study agree with previous literature, which has indicated that males are focused on long-term goals, reflecting an adaptive choice of long-term advantageous decks, while females are characterized by an exploratory approach ranging between short- and long-term consequences and are characterized by a more frequent selection of disadvantageous decks [39,46].

When the interaction between sex and weight condition was analyzed, females with overweight and normal weight did not differ in the long-term consequence index on the IGT, while males with overweight showed worse performance than males with normal weight. Different explanations may explain these different patterns. One possible explanation can be ascribed to the role of the central autonomic network (CAN; [47]), which involves the insula, ventromedial prefrontal cortex (vmPFC), and other cortical areas (e.g., cingulate cortex, sensorimotor cortices) in influencing the performance on executive tasks, including decision-making tasks [32,48]. The CAN controls and modulates autonomic activation (both sympathetic and parasympathetic branches) and central brain activation, influencing cognitive activities, especially executive functioning performance. The CAN differs between males and females due to metabolic and hormonal differences that characterize them [22]. Taken together with Damasio’s somatic marker hypothesis, which suggests a central role of the vmPFC in modulating the ability to make decisions [32], this aspect could suggest that overweight status in males is associated with an imbalance of CAN activation, which generates an impairment in the ability to evaluate long-term consequences of a choice adaptively. However, no studies have specifically focused on the role of the CAN in overweight status and obesity, and further studies are needed to highlight the direction of this association.

Considering that an alteration in decision making can represent a possible marker of weight gain, the different patterns of males and females could indicate that complex executive functions could influence eating behavioral risk factors associated with obesity in males. In females, other aspects appear to influence the occurrence of obesity, such as social expectations and stereotypes of body image [43].

Another explanation for the findings of this study may lie in the sex differences in the activation of the reward system associated with overeating and responses to food cues [49]. fMRI studies have demonstrated that females are characterized by hyperactivation of striate-limbic and frontal-cortical regions in response to food cues, independent of their weight condition, while males show a decreased activation in the middle frontal gyrus (associated with decision-making performance), insula, and cerebellum in response to food assumption [20]. These differences, manifested in decision-making performance, could justify the behavioral differences in approach to food and the different prevalence of overweight status and obesity, considering sex. However, how these neural differences are manifested behaviorally in obese and overweight populations remains unclear.

When studies have analyzed planning, a higher executive function useful for organizing and controlling complex behaviors (e.g., eating habits; [6]), no differences have emerged, whether considering weight conditions or the sex or the interaction between these variables. The association between planning and excessive body weight [30,50] is little examined and has yielded inconsistent results. Quavam and colleagues [30], analyzing a group of adolescents, found worse performance in the Tower of London (TOL) task, a measure of planning and problem solving, in adolescents with overweight status and obesity compared to normal weight. However, the authors did not compare overweight and obese adolescents. In contrast, Sweat et al. [50] did not find a difference between young adults with obesity and those with normal weight in planning abilities assessed by the TOL. To our knowledge, other studies have not analyzed planning performances in individuals with overweight compared to normal weight. In agreement with Sweat and colleagues [50], our study did not observe significant differences in TOL performance due to weight status.

Generally, the results of this study should be interpreted by considering different aspects. Unlike simple executive functions, which could represent a marker of the risk of weight gain, the more complex and integrated executive functions may come into play in a more complex way in overweight conditions, and they can be characterized by a bidirectional relationship with overweight status [6]. We can hypothesize that some executive functions (e.g., shifting, inhibition [51]) can represent risk factors for establishing maladaptive behaviors that lead to increased weight independently from other variables such as sex. In contrast, complex cognitive dimensions, such as decision making, are associated with weight conditions differently in males and females.

The absence of a general effect of the overweight condition would indicate that executive functions characterized by greater integration of neural networks (e.g., planning and decision making) can be associated with excessive body weight in a complex way involving bidirectional interactions [6,30]. Obesity appears to be related to many brain changes that potentially impact cognitive and executive functions [52], and the worsening of these abilities will exacerbate inappropriate behaviors causing obesity [53].

Although the preliminary results of this study allow for interesting considerations, some limitations should be highlighted. First, the sample size was relatively small, which may have prevented highlighting significant differences, limiting the generalizability of results. Another limitation is the cross-sectional design. A longitudinal study would highlight a possible trend in the relationship over time. The poor theoretical background of the study represents another limitation, specifically considering planning, that could have precluded the possibility of developing new inferences about the construct associated with weight condition. A further suggestion could be to consider cognitive tasks involving food cues in future studies in order to identify a possible involvement of high executive functions in response to food cues, rather than a general impairment, in people with moderate overweight conditions. Moreover, further studies should deepen exploration into the role of metabolic, hormonal, and neurochemical differences between males and females in influencing executive functioning and consequent goal-directed behaviors associated with overweight status and its exacerbation in obesity. Finally, although the selection of healthy participants prevented the risk of including possible confounding variables associated with health issues in overweight (e.g., hypertension, metabolic syndrome, eating disorders), further studies should consider including other psychological and physiological variables when comparing the groups.

## 5. Conclusions

Investigating the individual aspects that could influence eating behavior and body weight changes appears relevant, considering the role of obesity as a current public health concern. Knowing the role of some specific executive functions in driving complex behaviors, such as eating behavior, can encourage the consideration of body weight changes from a new perspective that allows the inclusion of cognitive variables in weight gain prevention programs. Potentially, these variables could influence people’s approach to food, thus influencing body condition. Although there have been few investigations on this topic, studies on weight loss interventions have emphasized the potential influences of executive functions on the success of these programs [6]. Understanding which executive functions are involved in overweight conditions and how males and females express them differently will allow new treatment approaches that integrate weight loss programs and executive functions training [6]. Moreover, such understanding can help us develop an integrated and more suitable theoretical model of the relationship between executive functions and excessive body weight.

## Figures and Tables

**Table 1:** Characteristics of the sample, classified according to age range and body mass index status.

Males			Females			
	Normal Weight	Overweight	Normal Weight	Overweight	F	*p*
N	14	18	15	18		
Age (mean, sd)	22.93 (2.56)	25.53 (2.72)	22.89 (1.53)	23.28 (2.47)	3.62	0.07
Years of Education (mean, sd)	16.00 (1.79)	15.73 (2.52)	16.83 (1.20)	16.72 (1.60)	<1	0.86
Physiological Measures (mean, sd)						
Weight (kg)	67.29 (7.54)	86.27 (12.41)	57.42 (7.76)	74.18 (7.78)	<1	0.63
Height (m)	1.76 (0.06)	1.79 (0.09)	1.67 (0.07)	1.67 (0.09)	<1	0.41
BMI	21.50 (1.65)	27.07 (2.31)	20.56 (2.04)	26.56 (1.82)	<1	0.66
Waist-to-Height Ratio	0.46 (0.04)	0.50 (0.05)	0.44 (0.04)	0.50 (0.05)	<1	0.32
Body Adiposity Index	24.10 (2.97)	27.25 (3.99)	26.06 (4.40)	33.56 (3.90)	4.45	0.04
Systolic Blood Pressure	127.29 (7.21)	125.54 (6.83)	113.17 (8.98)	114.06 (10.07)	<1	0.55
Diastolic Blood Pressure	73.50 (7.40)	74.77 (7.31)	72.28 (8.92)	72.00 (7.11)	<1	0.70

Note: N: number; F: Fisher’s F; *p*: significance; sd: standard deviation; BMI: Body Mass Index.

**Table 2:** Means and Standard Deviations of the groups in the IGT and TOL tasks.

Males			Females			
	Normal Weight	Overweight	Normal Weight	Overweight	F	*p*
IGT						
Learning of Long-Term Consequences (LTC)	21.43 (28.38)	−1.07 (22.66)	−6.11 (17.40)	2.94 (14.91)	8.97	0.004 *
Bias of Infrequent Loss (IFL)	0.86 (31.77)	−1.20 (16.90)	−0.78 (12.74)	0.47 (15.35)	<1	0.99
TOL						
Total Score	25.29 (8.04)	22.62 (6.89)	24.50 (4.96)	21.83 (8.60)	<1	0.94

* Significance level *p* < 0.05.

## Data Availability

Not Applicable.
